# Hypertensive disorders of pregnancy: heterogeneity in risk factors and maternal and neonatal outcomes across subtypes—a 6–year retrospective cohort study

**DOI:** 10.3389/fcvm.2026.1781870

**Published:** 2026-08-03

**Authors:** Li Jing, Li Dan, Mo Jiacheng, Mo Li, Zhang Chun, Zhang Jihong, Lu Yanqun, Wu Hua, Liang Xuxia

**Affiliations:** 1 Department of Obstetrics, Guangxi Medical Academy & Guangxi Zhuang Autonomous Region People’s Hospital, Nanning, China; 2 Department of Information, Guangxi Medical Academy & Guangxi Zhuang Autonomous Region People’s Hospital, Nanning, China

**Keywords:** chronic hypertension, cohort study, gestational hypertension, hypertensive disorders of pregnancy, maternal–neonatal outcomes, preeclampsia

## Abstract

**Background:**

Hypertensive disorders of pregnancy (HDP) are major obstetric complications that seriously threaten maternal and neonatal health. Current research predominantly focuses on preeclampsia (PE). However, systematic comparisons across all four HDP subtypes—gestational hypertension (GH), preeclampsia (PE), chronic hypertension (CH), and superimposed preeclampsia (SPE) remain limited, hindering a deeper understanding of their heterogeneous risks and precise management.

**Objective:**

This study aimed to systematically analyze the epidemiological trends, independent risk factors, and differential impacts on maternal and neonatal outcomes of HDP and its subtypes, based on large–sample clinical data.

**Methods:**

This retrospective cohort study was conducted at a single tertiary care center in southern China, including 20,081 pregnant women who delivered at Guangxi Zhuang Autonomous Region People's Hospital between January 2019 and December 2024. Participants were classified into an HDP group (*n* = 1,334) and a non–HDP group (*n* = 18,747) based on discharge diagnoses. The HDP group was further subdivided into GH, PE, CH, and SPE. Baseline characteristics and pregnancy outcomes were collected. Multinomial logistic regression was used to identify factors associated with each HDP subtype vs. non-HDP. Multivariate logistic regression was used to assess associations between HDP subtypes and adverse outcomes, and adjusted models were constructed to assess independent risks. Given the small SPE sample (*n* = 24), CH and SPE were combined (CH-SPE) for multivariable analyses to ensure model stability. Sensitivity analyses separating CH and SPE were also performed to assess the robustness of findings.

**Results:**

From 2019 to 2024, the overall incidence of HDP was 6.6%, with CH incidence increased by 78.2% (from 0.6% to 1.0%), and SPE incidence increased from 0.1% in 2019 to 0.7% in 2024. Multinomial analysis identified advanced maternal age (≥35 years), pre–pregnancy overweight/obesity, elevated fasting blood glucose (FBG), and gestational diabetes mellitus (GDM) as common risk factors for all HDP subtypes. Significant heterogeneity was observed among subtypes: PE demonstrated the strongest association with twin pregnancy (*OR*=4.071, 95% *CI*: 2.843––5.828), and elevated serum creatinine (Cr) was a specific risk factor for PE. CH–SPE was significantly associated with assisted reproductive technology (ART) (*OR*=2.032, 95% *CI*: 2.278––3.230), while higher education level was a unique protective factor (Postgraduate vs. Junior high school or below: *OR*=0.413, 95% *CI*: 0.259––0.660). All HDP subtypes significantly increased the risks of cesarean delivery with CH–SPE group (adjusted *OR*=3.399, 95% *CI*: 2.408––4.798) having the highest risk. Adverse neonatal outcomes (LBW and SGA) were predominantly concentrated in the PE and CH–SPE subtypes.

**Conclusion:**

Significant heterogeneity exists in risk factors and maternal–neonatal outcomes across different HDP subtypes. This study highlights the rapidly increasing incidence and unique risk profiles of chronic hypertension–based subtypes, emphasizing the urgency for precise screening and management pre–pregnancy and during early gestation. The findings provide important evidence for subtype–stratified risk assessment and individualized clinical intervention in HDP.

## Highlights

- Large-scale cohort study to systematically compare all four HDP subtypes in a southern Chinese population.
- Identified subtype-specific risk profiles and outcome patterns.
- Revealed rapidly increasing incidence of chronic hypertension-related subtypes (CH and SPE) over a 6–year period.
- Provides evidence for subtype-stratified clinical management.

## 1 Introduction

Hypertensive disorders of pregnancy (HDP) are a group of diseases specific to pregnancy with hypertension as the main clinical manifestation, which seriously threaten maternal and neonatal health and are one of the leading causes of maternal and perinatal mortality worldwide (1, 2). Based on its pathogenesis and clinical manifestations, HDP is typically classified as gestational hypertension (GH), preeclampsia (PE), chronic hypertension (CH), and chronic hypertension with superimposed preeclampsia (SPE) (3). The reported global incidence of HDP ranges from 5% to 10% (1, 4). In recent years, with the increasing prevalence of risk factors such as advanced maternal age, obesity, diabetes, and the application of assisted reproductive technology, the incidence of HDP has been increasing (5, 6).

At present, most existing studies have focused on the risk factors and adverse pregnancy outcomes of preeclampsia (PE) (7). In China, a systematic review and meta–analysis reported an overall HDP prevalence of approximately 7.6%, with PE being the most common subtype (8). However, this meta–analysis primarily focused on estimating the overall prevalence and subtype distribution rather than systematically comparing risk factor profiles or adverse pregnancy outcomes across the four subtypes. Significant differences may exist among HDP subtypes in terms of pathogenesis, clinical progression, and maternal and neonatal outcomes. Therefore, in–depth exploration of the independent risk factors of each subtype and their heterogeneous effects on pregnancy outcomes is essential for early identification, risk stratification, and precision intervention for HDP.

To address these gaps, we conducted a retrospective cohort of 20,081 pregnant women at Guangxi Zhuang Autonomous Region People's Hospital from 2019 to 2024, systematically analyzed the epidemiological trends, risk factor characteristics, and maternal and neonatal outcome differences across HDP subtypes, aiming to provide evidence–based evidence for individualized management and prognosis assessment in clinical practice.

## 2 Materials and methods

### 2.1 Research design and subjects

This was a single-center retrospective cohort study. This study included pregnant women who had established perinatal health records in the first trimester (6––12 weeks of gestation), completed standardized prenatal examinations, and delivered at the Department of Obstetrics of Guangxi Zhuang Autonomous Region People's Hospital from January 2019 to December 2024. Participants were classified into two groups based on the discharge diagnosis on the medical record: the HDP group (*n* = 1,334) and the non–HDP group (*n* = 18,747). Referring to the Guidelines for the Diagnosis and Treatment of Hypertensive disorders in pregnancy (2020) (9) and the International Society for the Study of Hypertension in Pregnancy (ISSHP) (10), the HDP group was further divided into four subtypes: GH group (*n* = 421), PE group (*n* = 705), CH group (*n* = 184), and SPE group (*n* = 24).

Inclusion criteria included: (1) gestational age at delivery ≥28 weeks; (2) complete clinical data. Exclusion criteria included: (1) coexisting with severe cardiac, hepatic, and renal diseases; (2) autoimmune disease; (3) the fetus had a serious congenital malformation or chromosomal abnormality. A total of 20,081 pregnant women were included in the final cohort. The study protocol was approved by the Ethics Committee of Guangxi Zhuang Autonomous Region People's Hospital (Approval Number: KY–SY–2023–023). Due to the retrospective nature of the study, informed consent was waived, and all personal information was de-identified to protect privacy.

### 2.2 Data collection

Demographic and clinical characteristics were extracted from electronic medical records, including maternal age, ethnicity, pre-pregnancy body mass index (BMI), educational level, marital status, occupation, smoking history, alcohol use during pregnancy, and family history of hypertension (defined as hypertension in first-degree relatives).

Pre-pregnancy BMI was calculated as weight in kilograms divided by height in meters squared and categorized according to the World Health Organization (WHO) standards: less than 18.5 kg/m^2^ is underweight, 18.5––25.0 kg/m^2^ is normal, 25.0––30.0 kg/m^2^ is overweight, and ≥30.0 kg/m^2^ is obese (11).

Pregnancy-related conditions included parity, mode of conception (natural or assisted reproductive technology, ART), twin pregnancy, and diagnosis of gestational diabetes mellitus (GDM). GDM was diagnosed according to the International Association of Diabetes and Pregnancy Study Groups (IADPSG) criteria as adopted in the Chinese guidelines (12, 13).

The first-trimester biochemical indicators were measured from fasting venous blood collected at 6–12 weeks of gestation, including hemoglobin (Hb), aspartate aminotransferase (AST), alanine aminotransferase (ALT), serum creatinine (Cr), blood urea nitrogen (BUN), and fasting blood glucose (FBG).

### 2.3 Outcome definitions

Maternal outcomes were defined as follow: Oligohydramnios was defined as an amniotic fluid index (AFI) ≤ 5 cm or a maximum vertical pocket (MVP) depth ≤2 cm on the last prenatal ultrasound examination before delivery or recorded amniotic fluid volume <300 mL at delivery; Postpartum hemorrhage was defined as blood loss ≥500 mL for vaginal delivery or ≥1000 mL for cesarean section; Cesarean section was defined as any cesarean delivery regardless of indication; and postpartum anemia was defined as Hb <110 g/L at 48–72 h postpartum.

Neonatal outcomes included gestational age at delivery, neonatal gender, birth weight, preterm birth (delivered at 28 to <37 weeks), small for gestational age (SGA), low birth weight (LBW; birth weight <2500 g), neonatal asphyxia (5–minute Apgar score <7), and stillbirth (fetal death at ≥28 weeks of gestation). SGA was defined as a birth weight lower than the 10th percentile for the gestational age (14).

### 2.4 Statistical methods

Data analysis was performed using R 4.5.1. Normality was tested using the Shapiro–Wilk method. Normally distributed data were presented as mean ± standard deviation (x ± *s*¯), and one-way ANOVA was used for comparisons between groups. Non–normally distributed data were presented as median (interquartile range) [*M (P25, P75)*], and comparisons between groups were conducted using the Kruskal–Wallis H test. Count data were expressed as the number of cases (percentage) [*n* (%)], and comparisons between groups were conducted using the chi-square test or Fisher's exact test. *post hoc* pairwise comparisons were corrected using the Bonferroni method (*P* < 0.013).

Multinomial logistic regression was used to explore factors associated with each HDP subtype compared with the non-HDP group. The dependent variable had five categories (non-HDP, GH, PE, CH, SPE), with non-HDP as the reference. Given the smaller sample size of the SPE group (*n* = 24), CH and SPE were combined into a CH-SPE group for the primary multivariable analysis to ensure model stability. Independent variables included maternal age (<35 *vs*. ≥ 35 years), pre-pregnancy BMI (categorized), educational level, parity, twin pregnancy, ART, family history of hypertension, GDM, and first-trimester Hb, AST, Cr, and FBG. Results were expressed as odds ratio (*OR*) and 95% confidence interval (*95% CI*).

Logistic regression was used to evaluate the associations between HDP subtypes and adverse maternal and neonatal outcomes. Two models were constructed: (1) a crude model (unadjusted); and (2) an adjusted model, which adjusted for potential confounders selected based on clinical relevance and univariate analysis (*P* < 0.05). The adjustment sets varied by outcome: For maternal outcomes (cesarean section, postpartum anemia, oligohydramnios) and neonatal LBW: adjusted for maternal age, pre-pregnancy BMI, educational level, parity, twin pregnancy, ART, family history of hypertension, GDM, first-trimester Hb, Cr, FBG, and gestational age at delivery. For preterm birth and SGA, the same variables except delivery gestational age.

Sensitivity analyses were performed to assess the robustness of the findings: (1) separating CH and SPE (i.e., analyzing CH and SPE as distinct categories) in the multinomial logistic regression to evaluate whether the combined CH-SPE analysis masked subtype-specific effects; (2) excluding data from the year 2019 (which had a lower GH incidence) to recalculate temporal trends of HDP subtypes, presented in Supplementary Materials.

All statistical tests were two-sided tests, and *P* < 0.05 was considered statistically significant, except where Bonferroni correction was applied.

## 3 Results

### 3.1 Incidence and temporal trends of HDP and its subtypes

**Figure 1:**
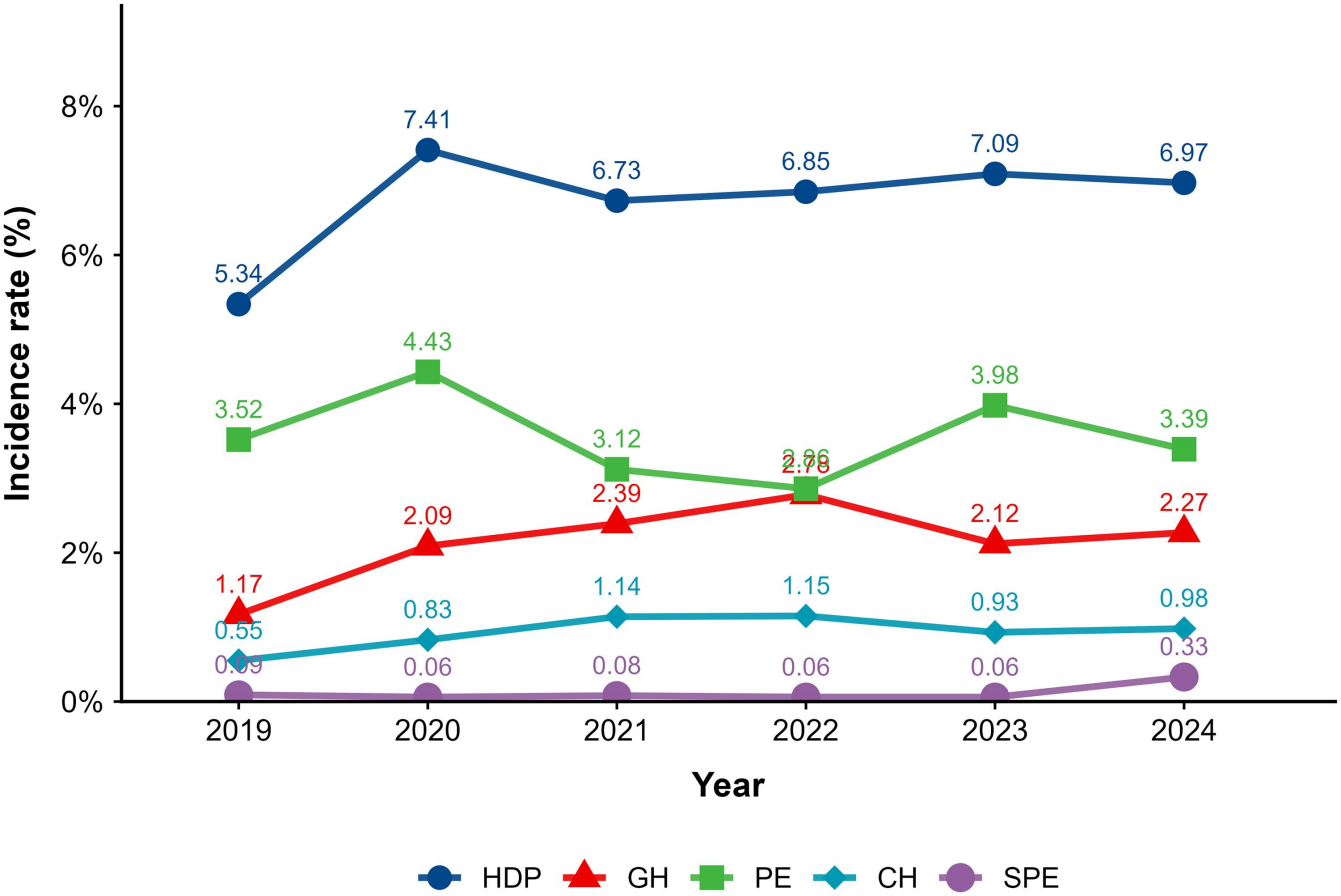
Incidence trends of HDP and its subtypes from 2019 to 2024.

Between 2019 and 2024, the overall incidence of HDP in this study cohort was 6.64% (1,334/20,081), with annual incidence ranging from 5.34% to 7.41%. There were significant differences in the incidence trends among subtypes: the incidence of GH was the lowest at 1.17% in 2019, then increased year by year to 2.78% in 2022, and then decreased slightly to 2.27% in 2024. The incidence of PE peaked at 4.43% in 2020, dropped to 2.86% in 2022, and rebounded to 3.39% in 2024. The incidence of CH continued to rise, increasing by 78.18% in 2024 compared to 2019. The incidence of SPE was at a stable low level (0.06%–0.09%) between 2019 and 2023, but rose significantly to 0.33% in 2024, an increase of about three times (Figure 1).

### 3.2 Baseline characteristics of HDP and Its subtypes

There were significant differences in multiple baseline characteristics between the HDP group and the non-HDP group. Compared with the non-HDP group, pregnant women in the HDP group were older, had a higher pre-pregnancy BMI, a higher proportion of multiparous women, and significantly higher proportions of ART, twin pregnancies, and a family history of hypertension (*P* < 0.001).

Heterogeneity in baseline characteristics was also observed across HDP subtypes. Pregnant women in the CH and SPE groups were older, had a higher pre–pregnancy BMI, and had a higher proportion of multiparity, ART, and a family history of hypertension. In terms of baseline biochemical indicators, Hb, AST, Cr, and FBG in the HDP group were significantly higher than those in the non–HDP group (*P* < 0.001). Subgroup analyses showed that the levels of these biochemical indicators in the PE, CH, and SPE groups were all higher than those in the GH group (all *P* < 0.05) (Table 1).

**Table 1:** Comparison of baseline characteristics between different subtypes of HDP and non–HDP pregnant women.

Characteristics	Non–HDP (*n* = 18,747)	HDP (*n* = 1,334)				*P*
		GH(*n* = 421)	PE(*n* = 705)	CH(*n* = 184)	SPE (*n* = 24)	
Baseline clinical features						
Age, years	31.52 ± ±4.46^bcde^	32.72 ± ±4.86^ade^	32.53 ± ±4.62^ade^	34.55 ± ±4.52^abc^	36.29 ± ±5.39^abc^	**<0**.**001**
<35 yr, *n* (%)	14100 (75.21)	267 (63.42)^ae^	469 (66.52)^ade^	94 (51.09)^ac^	10 (41.67)^abc^	**<0**.**001**
≥35 yr, *n* (%)	4647 (24.79)	154 (36.58)	236 (33.48)	90 (48.91)	14 (58.33)	
Ethnicity, *n* (%)						0.111
Han	10382 (55.38)^b^	197 (46.79)^a^	366 (51.91)	107 (58.15)	14 (58.33)	
Zhuang	7401 (39.48)	205 (48.69)	297 (42.13)	66 (35.87)	9 (37.50)	
Others	964 (5.14)	19 (4.51)	42 (5.96)	11 (5.98)	1 (4.17)	
Pre-pregnancy BMI, kg·m^–2^	22.31 ± ±3.35^bcde^	24.34 ± ±4.08^ade^	24.27 ± ±4.08^ade^	25.74 ± ±4.36^abce^	27.34 ± ±4.20^abcd^	**<0**.**001**
<18.5 kg/m^2^, n(%)	1777 (9.48)^bcd^	19 (4.51)^a^	33 (4.68)^a^	3 (1.63)^a^	0 (0.00)	**<0**.**001**
18.5∼25.0 kg/m^2^, n(%)	13528 (72.16)^bcde^	243 (57.72)^a^	403 (57.16)^a^	87 (47.28)^a^	8 (20.83)^a^	
25∼30 kg/m^2^, n(%)	3000 (16.00)^bcd^	115 (27.32)^a^	212 (30.07)^a^	66 (35.87)^a^	10 (41.67)	
≥30 kg/m, n(%)	442 (2.36)^bcde^	44 (10.45)^ad^	57 (8.09)^a^	28 (15.22)^ab^	9 (37.50)^a^	
Educational level						**<0**.**001**
Junior high school and below, *n* (%)	1141 (6.09)^d^	39 (9.26)	55 (7.80)^d^	30 (16.30)^ac^	4 (16.67)	
High school, *n* (%)	2738 (14.61)	61 (14.49)	105 (14.89)	21 (11.41)	3 (12.50)	
Undergraduate, *n* (%)	13210 (70.46)	285 (67.70)	495 (70.21)	118 (64.13)	11 (45.83)	
Postgraduate and above, *n* (%)	1658 (8.84)	36 (8.55)	50 (7.09)^e^	15 (8.15)	6 (25.00)^c^	
Marital status, *n* (%)						0.335
unmarried	1622 (8.65)	37 (8.79)	53 (7.51)	9 (4.89)	2 (8.83)	
Married	17125 (91.35)	384 (91.21)	652 (92.48)	175 (95.11)	22 (91.67)	
Occupation, *n* (%)						0.265
Professional skills	3951 (21.08)	96 (22.80)	150 (21.28)	42 (22.83)	10 (41.67)	
Service industry	9913 (52.87)	211 (50.12)	377 (53.48)	85 (46.20)	8 (33.34)	
Farmers	165 (0.88)	3 (0.71)	6 (0.85)	1 (0.54)	0 (0.00)	
Unemployed at home	4718 (25.17)	111 (26.37)	172 (24.40)	56 (30.43)	6 (25.00)	
Parity(time), *n* (%)						**<0**.**001**
0 time	9618 (51.30)^cd^	235 (55.82)	450 (63.83)^ade^	88 (47.83)^c^	11 (45.83)^c^	
1 time	7866 (41.96)^c^	162 (38.48)	227 (32.20)^a^	78 (42.39)	10 (41.67)	
≥2 times	1263 (6.74)^c^	24 (5.70)	28 (3.97)^ad^	18 (9.78)^c^	3 (12.50)	
ART, *n* (%)	1260 (6.72)^bcd^	46 (10.93)^a^	95 (13.48)^a^	29 (15.76)^a^	5 (20.83)	**<0**.**001**
Twin pregnancies, *n* (%)	390 (2.08)^bc^	20 (4.75)^ac^	68 (9.65)^ab^	9 (4.89)	1 (4.17)	**<0**.**001**
Smoking, *n* (%)	114 (0.61)	3 (0.71)	3 (0.43)	1 (0.54)	0 (0.00)	0.717
Drinking alcohol, *n* (%)	281 (1.50)	5 (1.19)	8 (1.13)	0 (0.00)	0 (0.00)	0.121
Family history of hypertension, *n* (%)	889 (4.74)^bcde^	35 (8.31)^a^	53 (7.52)^a^	19 (10.33)^a^	5 (20.83)^a^	**<0**.**001**
Baseline biochemical indicators [M(P25,P75)]						
Hb, g/L	125.00 (117.00–131.00)	126.00 (119.00–133.00)	126.50 (119.00–133.00)	130.00 (121.00–137.00)	131.50 (124.75–138.75)	**<0**.**001**
AST, U/L	11.00 (8.00–15.00)	11.00 (8.00–16.00)	12.00 (9.00–17.00)	12.00 (9.00–18.00)	9.00 (7.00–14.00)	**<0**.**001**
ALT·U/L	15.00 (13.00–18.00)	15.00 (13.00–18.00)	15.00 (13.00–18.00)	15.00 (13.00–17.75)	15.00 (12.50–18.00)	0.395
Cr, µmol/L	50.00 (45.00–55.00)	49.00 (44.00–54.00)	52.00 (47.00–57.00)	51.00 (46.00–55.00)	52.00 (46.50–57.00)	**<0**.**001**
BUN, mmol/L	2.60 (2.20–3.10)	2.60 (2.20–3.05)	2.60 (2.10–3.10)	2.50 (2.10–2.90)	2.80 (2.15–3.40)	0.086
FBG, mmol/L	4.65 (4.42–4.89)	4.68 (4.44–4.94)	4.70 (4.46–4.99)	4.77 (4.51–5.13)	4.85 (4.76–5.17)	**<0**.**001**
GDM at 24∼28 weeks	3605 (19.23)^bcd^	134 (31.83)^a^	207 (29.36)^a^	59 (32.07)^a^	9 (37.50)	<0.001

The *P–value* represents the result of comparing the HDP group as a whole with the non–HDP group. Bold values are used for visual emphasis and correspond to the superscript letters indicate significance based on *post hoc* pairwise comparisons (corrected *P* *<* 0*.*013): a compared with the non–HDP group; b compared with the GH group; c compared with PE; d compared with CH; e compared with SPE. HDP: Hypertensive disorders in pregnancy; GH: Gestational hypertension; PE: Preeclampsia; CH: Chronic hypertension with pregnancy; SPE: Chronic hypertension with preeclampsia. BMI: Body Mass Index; ART: Assisted reproduction technology; Hb: Hemoglobin; AST: Aspartate aminotransferase; ALT: Alanine aminotransferase; Cr: Serum creatinine; BUN: Serum urea nitrogen; FBG: Fasting blood glucose. GDM: Gestational diabetes mellitus.

### 3.3 Factors associated with HDP and Its subtypes

Multinomial logistic regression analysis revealed common risk factors as well as heterogeneous associations across HDP subtypes.

Common risk factors across all subtypes included advanced age (≥35 years), pre-pregnancy overweight/obesity, twin pregnancies, family history of hypertension, elevated levels of Hb and FBG in early pregnancy, and GDM were common risk factors for HDP and its subtypes.

Heterogeneous risk factors: PE showed the strongest association with twin pregnancies (*OR*=4.081,95% *CI*: 2.849–5.847), and elevated Cr levels (*OR*=1.012, 95% *CI*: 1.006–1.018) were a specific risk factor for it. In the primary analysis combining CH and SPE (CH-SPE group, *n* = 208), ART was significantly associated with CH-SPE(*OR* =  1.964, 95% *CI*: 1.197–3.224). Protective factor: Multiparity was a protective factor for CH and PE, except in the CH-SPE group. Higher educational attainment was a unique protective factor in the CH-SPE group, and the risk of CH-SPE decreased with higher educational attainment. Pregnant women with a postgraduate degree had an approximately 58% lower risk of CH-SPE compared with those with an education level below junior high school (*OR* =  0.413, 95% *CI*: 0.259–0.660) (Table 2).

**Table 2:** Multinomial logistic regression analysis of factors associated with HDP subtypes.

Variable	GH (*n* = 421)		PE (*n* = 705)		CH-SPE (*n* = 208)	
Age	OR	95% CI	OR	95% CI	OR	95% CI
<35 yr	1.000		1.000		1.000	
≥35 yr	1.834	1.418–2.372	1.572	1.284–1.925	1.957	1.373–2.790
Pre-pregnancy BMI						
<18.5 kg/m^2^	1.000		1.000		1.000	
18.5∼25.0 kg/m^2^	1.350	0.835–2.183	1.583	1.059–2.366	2.405	0.75–7.707
25.0∼30 kg/m^2^	2.424	1.450–4.052	3.072	2.012–4.689	6.911	2.133–22.389
≥30 kg/m^2^	3.739	2.061–6.783	6.681	4.216–10.588	17.003	5.119–56.474
Education level						
Junior high school and below	1.000		1.000		1.000	
High school	0.678	0.404–1.137	1.030	0.688–1.542	0.347	0.177–0.679
Undergraduate	0.661	0.383–1.142	0.676	0.429–1.067	0.499	0.258–0.966
Postgraduate and above	0.714	0.471–1.082	0.863	0.61–1.219	0.413	0.259–0.66
Parity time						
0 time	1.000		1.000		1.000	
1 time	0.696	0.541–0.895	0.509	0.417–0.622	0.909	0.633–1.305
≥2 times	0.433	0.247–0.758	0.372	0.239–0.580	0.796	0.416–1.524
Twin pregnancies	1.808	0.993–3.290	4.071	2.843–5.828	0.933	0.361–2.413
ART	1.100	0.742–1.630	1.055	0.787–1.413	2.032	1.278–3.230
Family history of hypertension	1.350	0.889–2.049	1.310	0.945–1.816	1.601	0.952–2.693
Baseline laboratory indicators						
Hb, g/L	1.006	0.995–1.016	1.010	1.002–1.018	1.036	1.019–1.052
AST, U/L	0.993	0.982–1.004	1.000	0.994–1.006	0.983	0.965–1.001
Cr, μmol/L	0.989	0.976–1.004	1.011	1.005–1.018	1.011	0.998–1.024
FBG, mmol/L	1.268	1.043–1.541	1.383	1.211–1.578	1.463	1.224–1.749
GDM	1.413	1.093–1.825	1.403	1.151–1.709	1.395	0.979–1.987

HDP, Hypertensive disorders in pregnancy; GH, Gestational hypertension; PE, Preeclampsia; CH, Chronic hypertension with pregnancy; SPE, Chronic hypertension with preeclampsia; BMI, Body Mass Index; ART, Assisted reproduction technology; Hb, Hemoglobin; AST, Aspartate aminotransferase; Cr, Serum creatinine; FBG, Fasting blood glucose; GDM, Gestational diabetes mellitus.

### 3.4 Pregnancy outcomes of HDP and its subtypes

The incidence of adverse maternal outcomes in the HDP group was significantly higher than that in the non-HDP group, particularly for cesarean section and postpartum anemia. All subtypes had significantly higher rates of cesarean section, especially in CH (77.2%) and SPE (83.3%), with higher rates of oligohydramnios and postpartum anemia in GH and PE. The impact of HDP on neonatal outcomes was mainly manifested in preterm birth, SGA, and LBW. The rates of preterm infants, SGA, and LBW were higher in the SPE and PE groups, and the average weight of newborns in the PE group was about 200 g lower than that in the non-HDP group. There were statistically significant differences in 1-minute Apgar scores among the groups, but no statistically significant differences in 5-minute, 10-minute scores and neonatal asphyxia rates. The incidence of stillbirth was low, with a total of 16 cases, including 15 (0.1%) in the non-HDP group and 1 (0.1%) in the PE group (Table 3).

**Table 3:** Comparison of pregnancy outcomes for HDP and its subtypes.

Outcomes	Non-HDP (*n* = 18747)	HDP (*n* = 1334)				*P*
		GH (*n* = 421)	PE (*n* = 705)	CH (*n* = 184)	SPE (*n* = 24)	
Maternal outcomes, *n* (%)						
Oligohydramnios	1899 (10.13)^bc^	66 (15.68)^a^	107 (15.18)^a^	19 (10.33)	5 (20.83)	<0.001
Postpartum hemorrhage	873 (4.66)	16 (3.80)	44 (6.24)	1 (0.54)	2 (8.33)	0.941
Postpartum anemia	7045 (37.58)^bc^	192 (45.61)^a^	323 (45.82)^a^	80 (43.48)	10 (41.67)	<0.001
Cesarean section	6928 (36.96)^bcde^	253 (60.10)^ad^	463 (65.67)^ad^	142 (77.17)^abc^	20 (83.33)^a^	<0.001
Placental abruption	385 (2.05)	8 (1.90)	21 (2.98)	1 (0.54)	2 (8.33)	0.434
Placenta previa	305 (1.63)	10 (2.38)	6 (0.85)	2 (1.09)	1 (4.17)	0.592
Neonatal outcomes						
Gestational age, weeks	39.12 ± 1.20^bcde^	38.77 ± 1.19^a^	38.46 ± 1.42^a^	38.23 ± 1.16^a^	38.05 ± 1.34^a^	<0.001
Preterm, *n* (%)	747 (3.90)^cd^	20 (4.54)^c^	87 (11.25)^ab^	17 (8.81)^a^	3 (12.00)	<0.001
Neonatal sex, *n* (%)						
Female	8944 (46.72)	226 (51.25)	377 (48.77)	81 (41.97)	14 (56.00)	0.149
male	10199 (53.28)	215 (48.75)	396 (51.23)	112 (58.03)	11 (44.00)	
Newborn weight, g	3188.46 ± 415.21^bcde^	3102.67 ± 477.53^ace^	2968.35 ± 536.30^abd^	3037.28 ± 461.87^ac^	2916.60 ± 630.48^ab^	<0.001
SGA, *n* (%)	1881 (9.83)^ce^	59 (13.38)^c^	161 (20.83)^abd^	20 (10.36)^c^	8 (32.00)^a^	<0.001
LBW, *n* (%)	836 (4.37)^bcde^	39 (8.84)^ac^	146 (18.89)^ab^	23 (11.92)^a^	6 (24.00)^a^	<0.001
Neonatal asphyxia, *n* (%)	194 (1.01)	4 (0.91)	12 (1.55)	2 (1.04)	1 (4.00)	0.282
Apgar score						
1-minute	9.86 ± 0.67^c^	9.84 ± 0.60	9.79 ± 0.80^a^	9.80 ± 0.62	9.68 ± 1.07	0.005
5-minutes	9.96 ± 0.38	9.96 ± 0.23	9.95 ± 0.46	9.94 ± 0.20	9.96 ± 0.20	0.395
10-minutes	9.98 ± 0.32	9.99 ± 0.08	9.97 ± 0.39	9.98 ± 0.12	10.00 ± 0.01	0.963
Stillbirth, *n* (%)	15 (0.08)	0 (0.00)	1 (0.13)	0 (0.00)	0 (0.00)	1.000

The *P*-value represents the result of the HDP group as a whole compared with the non–HDP group. Superscript letters indicate significance based on *post hoc* pairwise comparisons (corrected *P* *<* 0*.*013): a compared with the non–HDP group; b compared with the GH group; c compared with PE; d compared with CH; e vs. SPE. HDP, Hypertensive disorders in pregnancy; GH, Gestational hypertension; PE, Preeclampsia; CH, Chronic hypertension with pregnancy; SPE, Chronic hypertension with preeclampsia; SGA, Small for gestational age infants; LBW, Low birth weight infant.

### 3.5 Associations of HDP and its subtypes with adverse pregnancy outcomes

Multivariable logistic regression analyses showed that HDP was significantly associated with multiple adverse maternal and neonatal outcomes, with associations remaining robust after adjustment for confounders (Table 4).

**Table 4:** Logistic regression analysis of HDP and adverse maternal–neonatal outcomes.

Indicators	Crude model			Adjusted model		
	OR	95% CI	*P*	OR	95% CI	*P*
Maternal outcomes						
Oligohydramnios	1.537	1.312–1.801	<0.001	1.420	1.205–1.673	<0.001
Postpartum anemia	1.379	1.233–1.542	<0.001	1.260	1.124–1.414	<0.001
Cesarean section	3.285	2.922–3.692	<0.001	2.551	2.253–2.887	<0.001
Neonatal outcomes						
Preterm	2.212	1.771–2.762	<0.001	1.571	1.230–2.007	<0.001
SGA	2.040	1.760–2.364	<0.001	1.993	1.699–2.337	<0.001
LBW^a^	4.142	3.494–4.911	<0.001	2.771	2.213–3.470	<0.001

The data in the table are odds ratios (OR) and their 95% confidence intervals (95% CI). Crude model: unadjusted. Adjusted model: adjusted for maternal age, pre–pregnancy BMI, educational level, parity, twin pregnancy, ART, family history of hypertension, GDM, first–trimester Hb, Cr, FBG.

a LBW: further adjusted for delivery gestational age. SGA: Small for gestational age infants; LBW: Low birth weight infant.

**Figure 2:**
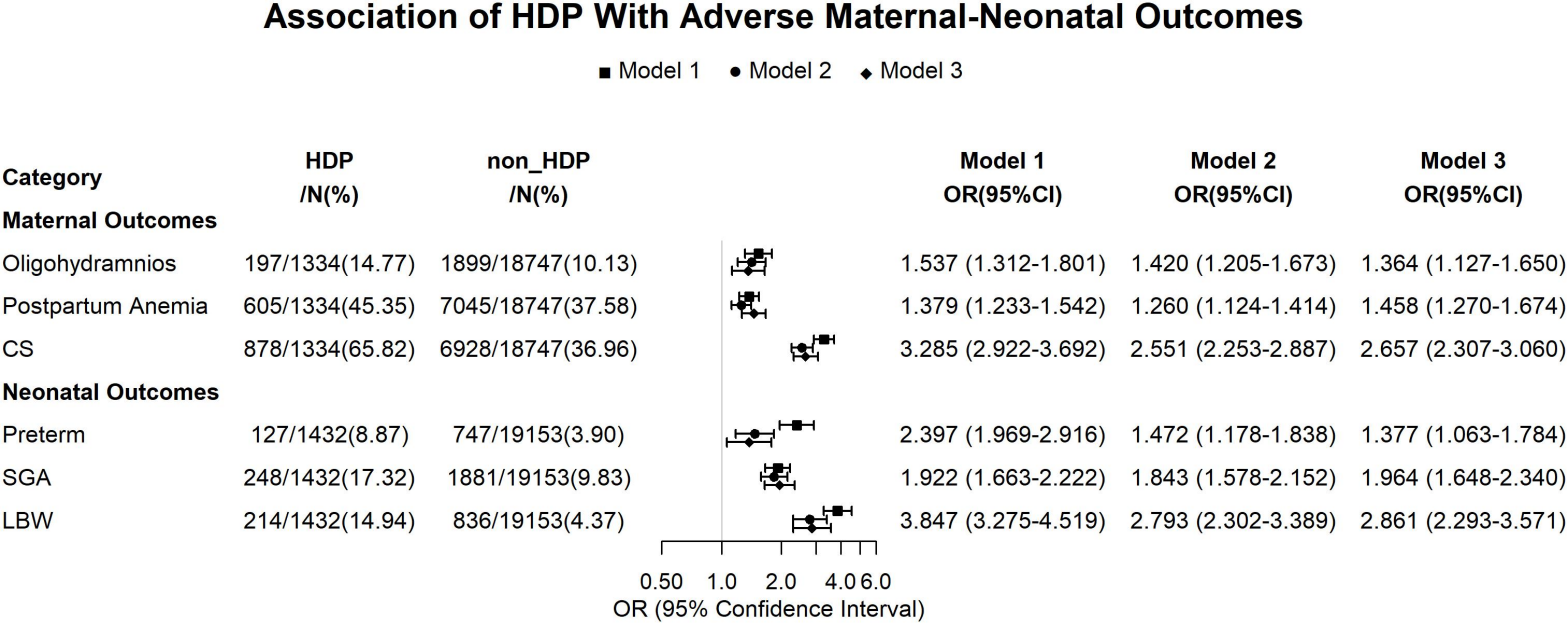
Forest plot of the association between HDP and adverse maternal-neonatal outcomes.

HDP (*vs.* non-HDP) was independently associated with increased risks of cesarean section (adjusted *OR*=2.551, 95% *CI*: 2.253–2.887), postpartum anemia (adjusted *OR*=1.260, 95% *CI*: 1.124–1.414), and oligohydramnios (adjusted *OR*=1.420, 95% *CI*: 1.205–1.673). Meanwhile, HDP significantly increased the risk of neonatal LBW (adjusted *OR* = 2.771,95% *CI*: 2.213–3.470), SGA (adjusted *OR* = 1.993, 95% *CI*: 1.699–2.337) and preterm birth (adjusted *OR* = 1.571,95% *CI*: 1.230–2.007) (Figure 2)

Multivariable logistic regression analysis revealed a significant heterogeneity in the associations between the subtypes of HDP and maternal and neonatal outcomes. All HDP subtypes significantly increased the risk of cesarean section, with the CH-SPE group having the highest risk (adjusted *O*  = 3.399, 95% *CI*: 2.408–4.798). The risk of postpartum anemia was mainly significant in the GH and PE groups. The risk of adverse neonatal outcomes (LBW and SGA) was highly concentrated in the PE and CH-SPE subtypes. PE was the strongest risk factor for SGA and LBW (SGA: *aOR* = 2.371,95% *CI*: 1.945–2.891; LBW: *aOR* = 3.419,95% *CI*: 2.600–4.496). Preterm birth risk was mainly associated with PE (*aOR* = 1.961,95% *CI*: 1.461–2.632) (Table 5).

**Table 5:** Logistic regression analysis of different subtypes of HDP and adverse maternal and neonatal outcomes.

Indicators	Crude model			Adjusted model		
	OR	95% CI	P	OR	95% CI	P
Maternal outcomes						
Oligohydramnios						
non-HDP	1.000			1.000		
GH	1.649	1.263–2.154	<0.001	1.515	1.154–1.989	0.003
PE	1.587	1.285–1.961	<0.001	1.404	1.130–1.745	0.002
CH-SPE	1.157	0.754–1.775	0.504	1.032	0.667–1.598	0.887
Postpartum anemia						
non–HDP	1.000			1.000		
GH	1.393	1.147–1.691	0.001	1.314	1.080–1.598	0.006
PE	1.404	1.208–1.634	<0.001	1.305	1.118–1.522	0.001
CH-SPE	1.267	0.961–1.669	0.093	1.143	0.863–1.514	0.352
Cesarean section						
non–HDP	1.000			1.000		
GH	2.569	2.109–3.129	<0.001	1.984	1.608–2.448	<0.001
PE	3.264	2.786–3.824	<0.001	2.314	1.950–2.745	<0.001
CH-SPE	6.008	4.325–8.347	<0.001	3.399	2.408–4.798	<0.001
Neonatal outcomes						
Preterm						
non–HDP	1.000			1.000		
GH	1.170	0.743–1.843	0.411	0.769	0.439–1.345	0.357
PE	3.123	2.470–3.950	<0.001	1.961	1.461–2.632	<0.001
CH-SPE	2.488	1.561–3.963	<0.001	1.851	1.067–3.211	0.029
SGA						
non–HDP	1.000			1.000		
GH	1.417	1.073–1.872	0.014	1.560	1.166–2.087	0.003
PE	2.414	2.017–2.890	<0.001	2.371	1.945–2.891	<0.001
CH–SPE	1.352	0.907–2.017	0.139	1.555	1.005–2.407	0.048
LBW^a^						
non-HDP	1.000			1.000		
GH	2.124	1.518–2.973	<0.001	1.726	1.107–2.689	0.016
PE	5.099	4.204–6.185	<0.001	3.419	2.600–4.496	<0.001
CH-SPE	3.360	2.259–4.998	<0.001	2.597	1.553–4.343	<0.001

The data in the table are odds ratios (OR) and their 95% confidence intervals (95% CI). Crude model: unadjusted. Adjusted model: adjusted for maternal age, pre-pregnancy BMI, educational level, parity, twin pregnancy, ART, family history of hypertension, GDM, first-trimester Hb, Cr, FBG.

a LBW: further adjusted for delivery gestational age. HDP: Hypertensive disorders in pregnancy; GH: Gestational hypertension; PE: Preeclampsia; CH-SPE: Chronic hypertension combined with pregnancy/preeclampsia combined group; SGA: Small for gestational age infants; LBW: Low birth weight infant.

### 3.6 Sensitivity analysis

Sensitivity analysis excluding 2019 data (Supplementary Figure S1) showed that the overall HDP incidence from 2020 to 2024 ranged from 6.9% to 7.4%, with no clear upward trend. The upward trends for CH and SPE were still observed but with reduced magnitudes, suggesting that the lower GH incidence in 2019 contributed to the apparent rapid increase in overall HDP rates when 2019 was included as the baseline.

Separating CH and SPE (Supplementary Table S2) revealed that the risk factor profiles for CH and SPE were generally consistent with the combined CH-SPE analysis, although the OR estimates for SPE had wider confidence intervals due to the small sample size. Notably, the association between ART and CH (*OR*=1.964, 95% *CI*: 1.197–3.224) remained significant, while the association with SPE was numerically higher but not statistically significant (*OR*=3.351, 95% *CI*: 0.908–12.368), likely reflecting insufficient statistical power rather than a true absence of effect. Separating CH and SPE revealed that the increased SGA risk in the combined CH-SPE group was primarily driven by SPE (*aOR* = 5.70), whereas CH mainly increased risks of preterm birth and LBW but not SGA, suggesting potentially distinct mechanisms affecting fetal growth (Supplementary Table S3).

## 4 Discussion

This large-scale retrospective cohort study systematically examined the heterogeneity in risk factors and maternal-neonatal outcomes across four HDP subtypes. Based on clinical data from 20,081 pregnant women, our findings not only confirm established risk factors for HDP but, more importantly, reveal distinct etiological and prognostic profiles for each subtype. The study highlights the rapidly increasing burden of chronic hypertension–related subtypes (CH and SPE) and identifies subtype–specific risk factors, such as twin pregnancy for PE and assisted reproductive technology for CH-SPE. These insights provide new evidence to guide subtype-stratified risk assessment and precision management in clinical practice.

### 4.1 Changes in the HDP disease spectrum: the burden of subtypes related to chronic hypertension becomes more prominent

The overall incidence of HDP in this study cohort was 6.6%, consistent (1, 4) with the globally reported range of 5% to 10%. A meta-analysis from China focused primarily on overall prevalence and subtype distribution rather than temporal trends or subtype-specific outcomes. Our study extends these national data by providing detailed annual incidence trends from 2019 to 2024, revealing that the increase in HDP incidence over this period was not uniform across subtypes. The incidence of CH continued to increase by 78.2% over six years (from 0.6% to 1.0%), while the incidence of SPE also increased from 0.1% in 2019 to 0.7% in 2024. This trend aligns with the global public health context of obesity among women of childbearing age, rising prevalence of metabolic syndrome, and delayed childbearing age (15, 16). The findings warn that the HDP subtype based on chronic hypertension is becoming an increasingly prominent clinical problem as the underlying health status of pregnant women changes, suggesting that obstetric clinicians need to move forward with the screening and management of chronic hypertension before and in the early stages of pregnancy.

### 4.2 Heterogeneity of risk factors: revealing different pathophysiological bases

Multivariate Logistic regression analysis indicated that although the subtypes of HDP “shared” some risk factors, there was significant heterogeneity in their risk factor profiles, which might reflect their different pathophysiological mechanisms.

First, advanced age, pre-pregnancy overweight/obesity, early pregnancy FBG, and mid-pregnancy GDM were common risk factors for all HDP subtypes. This finding is consistent with previous studies (5, 6), which have reported that age-related decline in vascular function and obesity-related chronic inflammatory state and insulin resistance are important bases (17, 18) for the occurrence of hypertension.

Secondly, the strongest association of PE with twin pregnancies (*OR* = 4.071) highlights the role of increased placental load (19). Notably, abnormal uterine spiral artery remodeling is a key pathophysiological feature of early-onset PE, whereas late-onset PE is more often associated with maternal metabolic and inflammatory factors (20). The association with twin pregnancy in our cohort may thus reflect the increased placental mass and higher risk of abnormal placentation, which are particularly relevant for early-onset PE. In addition, elevated Cr levels are a specific risk factor for PE, which directly confirms the disease nature of PE, characterized by involvement of terminal organs such as the kidneys (20). In the combined analysis of the CH-SPE group, assisted reproductive technology was a significant risk factor (*OR* = 2.032), often accompanied by multiple pregnancies, ovarian hyperstimulation, and underlying endocrine disorders, which may exacerbate endothelial dysfunction and cardiovascular stress (21) in patients with chronic hypertension. Sensitivity analysis separating CH and SPE (Supplementary Table S2) showed that the ART association remained significant for CH (*OR* = 1.964) but was numerically higher and non-significant for SPE (*OR* = 3.351, 95% *CI*: 0.908–12.368), likely due to the small SPE sample size rather than a true absence of effect.

In addition, the study found that educational attainment had a unique, gradient protective effect on CH-SPE. A population-based study in the United States found that the incidence of complications in pregnant women with CH was significantly higher among black people and Native Americans (22). Despite being a minority area with the largest Zhuang population in the country, there were no significant ethnic differences among HDP subtypes, suggesting a possible genetic background for different HDP subtypes, but higher health literacy could be achieved by improving chronic disease management, promoting healthy lifestyles, and enhancing medical compliance. It plays a key role in curbing the progression of chronic hypertension to more severe SPE and provides important evidence-based evidence for targeted health education interventions.

### 4.3 Differentiated disease burden in maternal and child outcomes: clinical practice guiding stratified management

The results of this study clearly show that the negative impact of HDP on mothers and children is not homogeneous but varies by subtype, which is crucial for clinical resource allocation and prognosis counseling.

On the maternal outcomes, all HDP subtypes significantly increased the risk of cesarean section, but the CH-SPE group had the highest risk of cesarean section (*OR* = 3.399), reflecting the complexity of the condition and more aggressive obstetric intervention in such patients. The risk of oligohydramnios and postpartum anemia was mainly concentrated in the GH and PE groups, which may be associated with the volume of blood in PE mothers and insufficient placental perfusion (23).

In terms of neonatal outcomes, adverse effects were highly concentrated in the PE and CH-SPE subtypes. PE was the strongest risk factor for preterm infants, SGA, and LBW, with an average neonatal weight reduction of about 200 g compared to the non-HDP group, reflecting the severe inhibition of fetal growth caused by PE placental insufficiency (24, 25). In contrast, the risk of neonatal outcome was relatively low in the GH group, and its association with preterm infants and LBW was not even significant after multifactorial adjustment. However, we caution against labeling GH as a “benign” condition. Due to the retrospective nature of this study, we were unable to determine how many women initially diagnosed with GH subsequently progressed to PE during pregnancy. Therefore, GH should not be considered entirely benign, and close monitoring for potential progression to PE remains warranted.

### 4.4 Strengths and limitations of the study

The strength of this study lies in its systematic parallel comparison and heterogeneity analysis of the four major subtypes of HDP based on a large sample of over 20,000 cases, overcoming the limitation of previous studies that mostly focused on PE. This study not only identified the unique risk patterns of each subtype but also quantified their differentiated outcome burden, providing a new and important perspective for the precise prevention and treatment of HDP.

The study also had several limitations. First, as a single-center retrospective study, there may be selection bias, and the extrapolation of the conclusions needs to be further verified by multi-center studies. Secondly, the sample size of the SPE group was small, and although we used a combined analysis to enhance statistical power, the in-depth exploration of its independent characteristics was still limited. The wide confidence intervals for SPE estimates in sensitivity analyses reflect this limitation. Future multi-center studies with larger SPE samples are needed. Third, due to the retrospective design, we were unable to collect several important clinical variables, including data on low-dose aspirin use for preeclampsia prevention among high-risk women, details on ART gamete donation, and longitudinal follow-up to determine how many women initially diagnosed with GH subsequently progressed to PE during pregnancy, which limits our ability to fully characterize the clinical trajectory of GH. Fourth, while we adjusted for delivery gestational age in most outcome models, residual confounding by indication (e.g., specific medication use during pregnancy and lifestyle details) cannot be entirely excluded. Additionally, the lack of postpartum long-term follow-up for cardiovascular and metabolic outcomes means our findings reflect only short-term effects and cannot capture the chronic disease impact of HDP. Fifth, we did not distinguish between early-onset and late-onset PE, which may have different underlying mechanisms (e.g., abnormal spiral artery remodeling is more characteristic of early-onset PE). This distinction could have provided deeper insights into the association between twin pregnancy and PE. Sixth, the exclusion of deliveries before 28 weeks of gestation may have introduced selection bias, as these very preterm deliveries often represent the most severe HDP phenotypes.

### 4.5 Conclusions and prospects

To sum up, this study systematically depicted the epidemiological, risk factor profiles, and maternal and fetal outcome risk maps of different subtypes of HDP through large sample cohort data, revealing their inherent heterogeneity. The findings emphasize that the management of HDP must shift from “homogenization” to “subtype stratification” strategies. In clinical practice, the focus should be on preventing SPE in pregnant women with chronic hypertension or undergoing ART; PE should be closely monitored in twin pregnancies; For those who have been diagnosed with PE and SPE, they should be the focus of intervention for improving neonatal outcomes. Prospective studies and in-depth mechanism exploration in the future will provide stronger evidence for further refined management and improvement of maternal and infant prognosis. Among emerging therapeutic avenues, placenta-targeted drug delivery systems represent a particularly promising frontier for HDP, especially for preeclampsia. By enabling localized, high-concentration drug delivery to the placenta while minimizing systemic and fetal exposure, these strategies could potentially address the underlying placental pathophysiology rather than merely managing maternal symptoms. Although still in early preclinical and clinical stages, this approach offers a paradigm shift toward targeted, disease-modifying therapies for HDP (26, 27).

## Data Availability

The raw data supporting the conclusions of this article will be made available by the authors, without undue reservation.
