# Supplementary material for: Hypertensive disorders of pregnancy: heterogeneity in risk factors and maternal and neonatal outcomes across subtypes—a 6–year retrospective cohort study
Source: Front Cardiovasc Med. 2026 Aug 3;13:1781870. doi: 10.3389/fcvm.2026.1781870 (PMC13478100; doi:10.3389/fcvm.2026.1781870)
Supplement: Supplementary file 1 [file Datasheet1.docx]

Online-only Supplementary Materials

Title：**Hypertensive Disorders of Pregnancy: Heterogeneity in Risk Factors and Maternal and Neonatal Outcomes Across Subtypes — A 6-Year Retrospective Cohort Study**

Li et al

Supplemental table 1: Multivariate logistic regression analysis of factors influencing HDP

| **Influencing factors** | **HDP (n=1334)** | | |
| --- | --- | --- | --- |
| **Age** | ***OR*** | ***95%CI*** | ***P* value** |
| <35 yr | 1.000 |  |  |
| ≥35 yr | 1.707 | 1.469-1.984 | <0.001 |
| **Pre-pregnancy BMI** | |  |  |
| <18.5 kg·m^-2^ | 1.000 |  |  |
| 18.5 ~ 25.0 kg·m^-2^ | 1.613 | 1.196-2.174 | 0.002 |
| 25.0 ~ 30 kg·m^-2^ | 4.050 | 2.942-5.576 | <0.001 |
| ≥30 kg·m^-2^ | 6.286 | 4.158-9.502 | <0.001 |
| **Education level** |  |  |  |
| Junior high school and below | 1.000 |  |  |
| High school | 0.747 | 0.556-1.002 | 0.052 |
| College | 0.609 | 0.442-0.839 | 0.002 |
| Postgraduate and above | 0.695 | 0.545-0.885 | 0.003 |
| **Parity time** |  |  |  |
| 0 time | 1.000 |  |  |
| 1 time | 0.617 | 0.532-0.715 | <0.001 |
| ≥2 times | 0.442 | 0.323-0.606 | <0.001 |
| **Twin pregnancies** | 2.780 | 2.049-3.771 | <0.001 |
| **ART** | 1.186 | 0.952-1.477 | 0.128 |
| **Family history of hypertension** | 1.361 | 1.066-1.736 | 0.013 |
| **Baseline laboratory indicators** | |  |  |
| Hb, g/L | 1.012 | 1.006-1.018 | <0.001 |
| AST, U/L | 0.997 | 0.991-1.002 | 0.330 |
| Cr, umol/L | 1.007 | 1.001-1.012 | 0.022 |
| FBG, mmol/L | 1.380 | 1.235-1.543 | <0.001 |
| **GDM** | 1.397 | 1.203-1.621 | <0.001 |

Note: HDP: Hypertensive disorders in pregnancy; BMI: Body Mass Index; ART: Assisted reproduction technology; Hb: Hemoglobin; AST: Aspartate aminotransferase; Cr: Serum creatinine; FBG: Fasting blood glucose. GDM: Gestational diabetes mellitus.

**Supplemental Table 2 Multinomial logistic regression analysis of factors influencing its subtypes of HDP**

| **Influencing factors** | **GH (n=421)** | | **PE (n=705)** | | **CH (n=184)** | | **SPE(n=24)** | |
| --- | --- | --- | --- | --- | --- | --- | --- | --- |
|  | ***OR*** | ***95%CI*** | ***OR*** | ***95%CI*** | ***OR*** | ***95%CI*** | ***OR*** | ***95%CI*** |
| **Age** |  |  |  |  |  |  |  |  |
| <35 yr | 1.000 |  | 1.000 |  | 1.000 |  | 1.000 |  |
| ≥35 yr | 1.855 | 1.434-2.399 | 1.582 | 1.292-1.936 | 2.117 | 1.459-3.073 | 0.952 | 0.295-3.075 |
| **Pre-pregnancy BMI** |  |  |  |  |  |  |  |  |
| <18.5 kg·m^-2^ | 1.000 |  | 1.000 |  | 1.000 |  | 1.000 |  |
| 18.5 ~ 25.0 kg·m^-2^ | 1.410 | 0.875-2.273 | 1.658 | 1.112-2.473 | 2.440 | 0.763-7.803 | 110.715 | 44.617-274.73 |
| 25.0 ~ 30 kg·m^-2^ | 2.738 | 1.618-4.634 | 4.276 | 2.795-6.543 | 8.181 | 2.507-26.669 | 802.678 | 335.812-1918.606 |
| ≥30 kg·m^-2^ | 4.885 | 2.454-9.726 | 5.488 | 3.135-9.605 | 17.837 | 5.034-63.203 | 759.251 | 193.42-2980.365 |
| **Education level** |  |  |  |  |  |  |  |  |
| Junior high school and below | 1.000 |  | 1.000 |  | 1.000 |  | 1.000 |  |
| High school | 0.682 | 0.406-1.144 | 1.027 | 0.687-1.537 | 0.328 | 0.160-0.671 | 0.683 | 0.098-4.779 |
| College | 0.660 | 0.382-1.141 | 0.660 | 0.418-1.040 | 0.433 | 0.213-0.880 | 1.464 | 0.227-9.421 |
| Postgraduate and above | 0.718 | 0.473-1.090 | 0.849 | 0.601-1.200 | 0.418 | 0.257-0.682 | 0.456 | 0.093-2.235 |
| **Parity time** |  |  |  |  |  |  |  |  |
| 0 time | 1.000 |  | 1.000 |  | 1.000 |  | 1.000 |  |
| 1 time | 0.701 | 0.545-0.902 | 0.510 | 0.418-0.623 | 0.961 | 0.658-1.404 | 0.595 | 0.182-1.945 |
| ≥2 times | 0.434 | 0.247-0.760 | 0.369 | 0.237-0.574 | 0.773 | 0.364-1.476 | 1.344 | 0.245-7.374 |
| **Twin pregnancies** | 1.825 | 1.003-3.321 | 4.081 | 2.849-5.847 | 0.861 | 0.301-2.464 | 1.656 | 0.185-14.833 |
| **ART** | 1.111 | 0.749-1.647 | 1.043 | 0.777-1.399 | 1.964 | 1.197-3.224 | 3.351 | 0.908-12.368 |
| **Family history of hypertension** | 1.340 | 0.882-2.038 | 1.318 | 0.951-1.827 | 1.444 | 0.819-2.547 | 2.814 | 0.746-10.616 |
| **Baseline laboratory indicators** | | | | | | | | |
| Hb, g/L | 1.006 | 0.995-1.016 | 1.010 | 1.002-1.019 | 1.036 | 1.019-1.053 | 1.035 | 1.011-1.059 |
| AST, U/L | 0.994 | 0.983-1.005 | 1.001 | 0.995-1.007 | 0.989 | 0.971-1.006 | 0.906 | 0.813-1.009 |
| Cr, umol/L | 0.990 | 0.976-1.004 | 1.012 | 1.006-1.018 | 1.009 | 0.994-1.024 | 1.024 | 1.004-1.044 |
| FBG, mmol/L | 1.268 | 1.043-1.544 | 1.403 | 1.230-1.602 | 1.481 | 1.227-1.789 | 1.545 | 1.07-2.23 |
| **GDM** | 1.416 | 1.096-1.830 | 1.396 | 1.146-1.701 | 1.274 | 0.873-1.858 | 2.723 | 0.946-7.838 |

**Supplemental Table 3 Logistic regression analysis of different subtypes of HDP and adverse maternal and fetal outcomes**

| **Indicators** | **Crude Model** | | | **Adjusted Model** | | |
| --- | --- | --- | --- | --- | --- | --- |
|  | ***OR*** | ***95%CI*** | ***P*** | ***OR*** | ***95%CI*** | ***P*** |
| **Maternal outcomes** |  |  |  |  |  |  |
| **Oligohydramnios** |  |  |  |  |  |  |
| non-HDP | 1.000 |  |  | 1.000 |  |  |
| GH | 1.649 | 1.263, 2.154 | <0.001 | 1.516 | 1.155, 1.990 | 0.003 |
| PE | 1.587 | 1.285, 1.961 | <0.001 | 1.405 | 1.131, 1.745 | 0.002 |
| CH | 1.022 | 0.634, 1.646 | 0.930 | 0.915 | 0.563, 1.487 | 0.720 |
| SPE | 2.335 | 0.871, 6.260 | 0.092 | 2.043 | 0.744, 5.607 | 0.166 |
| **Postpartum anemia** |  |  |  |  |  |  |
| non-HDP | 1.000 |  |  | 1.000 |  |  |
| GH | 1.393 | 1.147, 1.691 | 0.001 | 1.313 | 1.080, 1.598 | 0.006 |
| PE | 1.404 | 1.208, 1.634 | <0.001 | 1.305 | 1.118, 1.522 | 0.001 |
| CH | 1.278 | 0.953, 1.713 | 0.101 | 1.156 | 0.859, 1.558 | 0.339 |
| SPE | 1.186 | 0.527, 2.673 | 0.680 | 1.042 | 0.459, 2.368 | 0.921 |
| **Cesarean section** |  |  |  |  |  |  |
| non-HDP | 1.000 |  |  | 1.000 |  |  |
| GH | 2.569 | 2.109, 3.129 | <0.001 | 1.984 | 1.608, 2.448 | <0.001 |
| PE | 3.264 | 2.786, 3.824 | <0.001 | 2.314 | 1.950, 2.745 | <0.001 |
| CH | 5.768 | 4.083, 8.149 | <0.001 | 3.343 | 2.327, 4.802 | <0.001 |
| SPE | 8.530 | 2.914, 24.966 | <0.001 | 3.963 | 1.302, 12.057 | 0.015 |
| **Neonatal outcomes** |  |  |  |  |  |  |
| **Preterm** |  |  |  |  |  |  |
| non-HDP | 1.000 |  |  | 1.000 |  |  |
| GH | 0.981 | 0.573, 1.681 | 0.945 | 0.769 | 0.439, 1.345 | 0.357 |
| PE | 2.946 | 2.259, 3.842 | <0.001 | 1.961 | 1.461, 2.633 | <0.001 |
| CH | 2.349 | 1.354, 4.074 | 0.002 | 1.822 | 1.014, 3.274 | 0.045 |
| SPE | 2.593 | 0.608, 11.051 | 0.198 | 2.079 | 0.447, 9.669 | 0.351 |
| **SGA** |  |  |  |  |  |  |
| non-HDP | 1.000 |  |  | 1.000 |  |  |
| GH | 1.508 | 1.140, 1.993 | 0.004 | 1.561 | 1.167, 2.089 | 0.003 |
| PE | 2.628 | 2.186, 3.160 | <0.001 | 2.373 | 1.946, 2.893 | <0.001 |
| CH | 1.065 | 0.661, 1.717 | 0.796 | 1.212 | 0.734, 2.000 | 0.453 |
| SPE | 3.809 | 1.577, 9.197 | 0.003 | 5.696 | 2.235, 14.514 | <0.001 |
| **LBW*** |  |  |  |  |  |  |
| non-HDP | 1.000 |  |  | 1.000 |  |  |
| GH | 2.108 | 1.466, 3.031 | <0.001 | 1.728 | 1.190, 2.545 | 0.016 |
| PE | 5.657 | 4.614, 6.935 | <0.001 | 3.422 | 2.604, 4.155 | <0.001 |
| CH | 3.366 | 2.143, 5.287 | <0.001 | 2.320 | 1.332, 4.039 | 0.003 |
| SPE | 6.523 | 2.429, 17.518) | <0.001 | 5.747 | 1.626, 20.308 | 0.007 |

Note: The data in the table are odds ratios (OR) and their 95% confidence intervals (95% CI). Crude model: unadjusted. Adjusted model: adjusted for maternal age, pre-pregnancy BMI, educational level, parity, twin pregnancy, ART, family history of hypertension, GDM, first-trimester Hb, Cr, FBG. *LBW: further adjusted for delivery gestational age. HDP: Hypertensive disorders in pregnancy; GH: Gestational hypertension; PE: Preeclampsia; CH-SPE: Chronic hypertension combined with pregnancy/preeclampsia combined group; SGA: Small for gestational age infants; LBW: Low birth weight infant.

**Supplement Table 4. Comparison of Diagnostic Criteria for Hypertensive Disorders of Pregnancy (HDP) Between the Chinese Guideline (2020) and the ISSHP Guideline (2018)**

| **Diagnostic Criteria** | **Chinese Guideline (2020)** | **ISSHP Guideline (2018)** | **Consistency** |
| --- | --- | --- | --- |
| **Hypertension threshold** | SBP ≥140 mmHg and/or DBP ≥90 mmHg (two measurements ≥4 hours apart) | SBP ≥140 mmHg and/or DBP ≥90 mmHg (two measurements ≥4 hours apart) | **Identical** |
| **Gestational Hypertension (GH)** | New-onset hypertension after **20 weeks** of gestation without proteinuria | New-onset hypertension after **20 weeks** of gestation without proteinuria | **Identical** |
| **Preeclampsia (PE)** | New-onset hypertension after 20 weeks with **proteinuria (≥0.3g/24h or ≥+ on dipstick)** OR end-organ dysfunction without proteinuria | New-onset hypertension after 20 weeks with **proteinuria (≥0.3g/24h or PCR ≥30 mg/mmol)** OR maternal end-organ/placental dysfunction without proteinuria | **Highly consistent** (minor differences in proteinuria measurement) |
| **Chronic Hypertension (CH)** | Hypertension present **before pregnancy or before 20 weeks** of gestation, or persisting **>12 weeks postpartum** | Hypertension present **before pregnancy or before 16 weeks** of gestation, or persisting **>6 weeks postpartum** | **Highly consistent** (minor differences in time cutoffs) |
| **Chronic Hypertension with Superimposed PE (SPE)** | New-onset or worsening proteinuria, OR abrupt increase in BP, OR other features of PE in a woman with CH | New-onset or worsening proteinuria, OR sudden increase in BP (requiring urgent therapy), OR other features of PE in a woman with CH | **Identical** |

**Abbreviations:** SBP, systolic blood pressure; DBP, diastolic blood pressure; PCR, protein-to-creatinine ratio; ISSHP, International Society for the Study of Hypertension in Pregnancy; GH, gestational hypertension; PE, preeclampsia; CH, chronic hypertension; SPE, chronic hypertension with superimposed preeclampsia.

**Source of ISSHP criteria:** Brown MA, Magee LA, Kenny LC, et al. Hypertensive Disorders of Pregnancy: ISSHP Classification, Diagnosis, and Management Recommendations for International Practice. *Hypertension*. 2018;72(1):24-43. doi:10.1161/HYPERTENSIONAHA.117.10803.

**Source of Chinese criteria:** Hypertensive Disorders in Pregnancy Group, Chinese Society of Obstetrics and Gynecology. Guidelines for Diagnosis and Treatment of Hypertensive Disorders in Pregnancy (2020). *Chinese Journal of Obstetrics and Gynecology*. 2020;55(4):227-238. doi:10.3760/cma.j.cn112141-20200114-00039.

Supplementary Figure S1 Incidence trends of HDP and its subtypes from 2020 to 2024
